# Virulence Properties of GI-23 Infectious Bronchitis Virus Isolated in Poland and Efficacy of Different Vaccination strategies

**DOI:** 10.3390/pathogens10050522

**Published:** 2021-04-26

**Authors:** Anna Lisowska, Anna Pikuła, Justyna Opolska, Agnieszka Jasik, Anna Kycko, Katarzyna Domańska-Blicharz

**Affiliations:** 1 Department of Poultry Diseases, National Veterinary Research Institute, Partyzantów 57, 24-100 Puławy, Poland; anna.lisowska@piwet.pulawy.pl (A.L.); justyna.opolska@piwet.pulawy.pl (J.O.); 2 Department of Pathology, National Veterinary Research Institute, Partyzantów 57, 24-100 Puławy, Poland; agnieszka@piwet.pulawy.pl (A.J.); anna.kycko@piwet.pulawy.pl (A.K.)

**Keywords:** infectious bronchitis virus (IBV), GI-23 strain, pathogenicity, cross-protection

## Abstract

Infectious bronchitis virus (IBV) is one of the most important poultry pathogens, leading significant economic losses worldwide. IBV is characterised by highly genetic, serotype, and pathotypic variability. Despite extensive immunoprophylaxis strategies, the emergence of new genetic lineages is frequently observed in the field, causing disease control to be more complicated. In the last decade, the spread of variants assigned to the GI-23 lineage of IBV (formerly known as Var2) started from Middle-Eastern countries and reached Europe in the last few years. Recently, the introduction and fast spread of Var2-like IBVs in Poland was reported. In this study, the virulence properties and efficacy of different vaccination programmes were evaluated against infection with the IBV GI-23 strain gammaCoV/Ck/Poland/G052/2016. The pathogenicity of the Var2 isolate was conducted in one-day-old and three-week-old SPF chickens and showed that the course of the disease is age dependent. Seven vaccination programmes using Mass, 793B, QX alone or in combination, and Var2 live vaccines were tested against the GI-23 infectious bronchitis virus challenge. All groups were scored according to the ciliostasis test at 5 days post challenge. Two immunoprophylaxis strategies generated full protection against gammaCoV/Ck/Poland/G052/2016 infection—Var2 and Mass used in one-day-old chickens boosted by a combination of the QX and 793B vaccine (both with a ciliostasis score of 0 and 100% protection).

## 1. Introduction

Infectious bronchitis virus (IBV) is the longest known coronavirus in the world as it was firstly described almost 90 years ago [1,2]. The virus causes a disease of chicken called infectious bronchitis, but also affects the reproductive and renal system, which contributes significant economic losses to the poultry industry. Despite years of intensive research, the encouragement of biosecurity on farms, the availability of many attenuated live and inactivated vaccines, and widespread chicken immunization, the disease continues to emerge. The main reason is the extraordinary variability of the virus, a consequence of belonging to the *Coronaviridae* family, which makes it exist in many genetic and serological variants [3]. The most variable part of the genome is that the protein encoding the spike protrudes above the surface of the virus (S1 coding region). The spike mediates cell attachment, virus-cell membrane fusion, plays an important role in host cell specificity, and contains epitopes that induce neutralizing antibodies in the chicken [4]. Nucleotide heterogeneity is most prevalent in the S1 portion of the S gene and is largely contained within three different hypervariable regions (HVRs) corresponding to amino acids 38–67, 91–141, and 274–387 (HVR1, HVR2, and HVR3, respectively) [5]. According to the most recent virus taxonomy, IBV belongs to the *Gammacoronavirus* genus, *Igacovirus* subgenus, and is represented by two ratified species: Avian coronavirus (AvCoV) and avian coronavirus 9203 (AvCoV9203), which include also turkey and guinea-fowl coronaviruses [6]. The unified IBV classification, based on the diversity of the S1 coding region, introduced in 2016, includes at least 32 different viral lineages arranged into six genotypes and a number of unique variants, but the identification of new genotypes/lineages was subsequently reported [5,7]. The occurrence of viruses of the GI-23 lineage formerly known as Var2 variants for over a dozen years was restricted to the region of the Middle East. At the end of 2015, the first case of infectious bronchitis caused by GI-23 (Var2) IBV was recognized in Poland [8]. Furthermore, the emergence of closely-related Var2 strains was reported in Romania and Germany, indicating the spread of this variant in Europe [9,10]. In Poland, in the following years, the number of Var2 cases had increased accounting for over 28% of all lineages detected between 2018–2019 [8,11]. Six months after the first IBV GI-23 disease outbreak, the registration of the homologous vaccine for this IBV lineage (Tabic IB Var206) was approved by the Polish veterinary authorities, which made a total of five virus types (GI-1 = Mass, GI-(1 + 12) = Mass + D274, GI-13 = 793B, and GI-19 = QX) contained in many live attenuated vaccines available in Poland, which are used in combination or alone.

The aim of this study was the assessment of pathogenicity of previously genetically characterised IBV GI-23 strain gammaCoV/Ck/Poland/G052/2016 in SPF chickens. Moreover, the efficacy of different vaccination programmes to the infection of this strain was also investigated.

## 2. Results

### 2.1. Pathogenicity Study in SPF Chickens

#### 2.1.1. Clinical Findings

**Figure 1:**
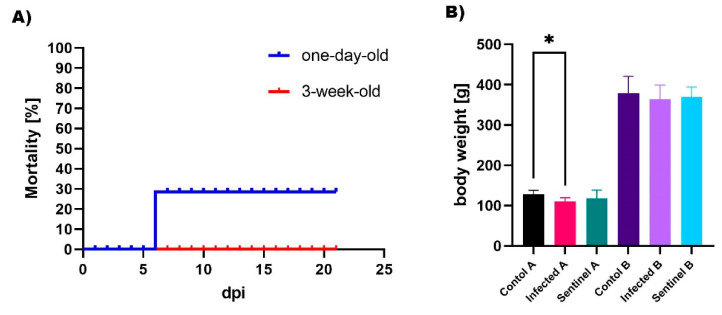
Comparison of selected parameters of clinical findings from a pathogenicity study. (**A**) Mortality curves of the challenged SPF chickens, each group was denoted. (**B**) Mean body weight gain of one-day-old (denoted as A group) and 3-week-old (group B) SPF chickens after infection with IBV gammaCoV/Ck/Poland/G052/2016 strain (* *p* < 0.05). During the experimental trial, the effect of infection with the gammaCoV/Ck/Poland/G052/2016 strain on chicken weight gain was evaluated. The observed lower body weight in infected 3-week-old birds (infected birds: AMn = 363.9 g, SD = 35.4; control birds AMn = 378.8 g, SD = 41.7) was not statistically significant (*p* = 0.54). In turn, in one-day-old birds analysis confirmed a statistically significant difference in the mean body weight (infected birds: AMn = 110.4 g; SD = 9.5; control birds: AMn = 128.1 g; SD = 9.8) (Figure 1B) between infected and control groups.

The virulence properties were assessed using SPF chickens in two different age groups. On the second day after infection, one-day-old birds showed symptoms of the disease, such as depression (*n* = 5), ruffled feathers (*n* = 4), thermoregulation disorders (huddling) (*n* = 4), respiratory symptoms (*n* = 3), and diarrhoea (*n* = 4). Four days post infection (dpi), exacerbation of respiratory (tracheal rales) (*n* = 7) and intestinal (dark-brown wet droppings) (*n* = 9) signs were observed. A total of three chickens died at 6 dpi resulting in 30% mortality (Figure 1A). From the second week of observation, the symptoms were less severe, all chickens had diarrhea and individual birds showed mild respiratory signs (*n* = 3), which persisted until the end of clinical observation. Similar clinical symptoms also appeared in sentinel chickens. For the three-week-old SPF chickens, at 2 dpi only mild diarrhoea (*n* = 10) was present and since 10 dpi birds did not show any clinical signs. No clinical symptoms were observed in both age control groups.

#### 2.1.2. Pathological Findings

**Figure 2:**
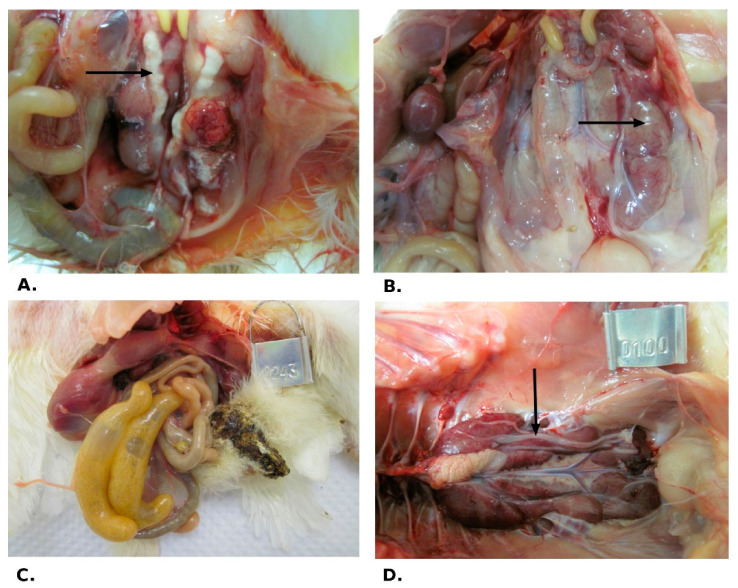
Macroscopic changes observed at necropsy during pathogenicity study. (Trial 1: (**A**–**C**), trial 2: (**D**)) (**A**). presence of urate in the ureter in a dead bird (6 dpi); (**B**). pale, swollen kidneys in a dead bird (6 dpi); (**C**). bloated and filled with yellowish content intestinal tracts of chicken at 14 dpi; and (**D**). presence of urate in the ureter, oedema, and renal congestion in a bird sectioned at 21 dpi. Black arrows indicate lesions detected in tissue.

In trial 1, the pathological changes were observed both in dead (6 dpi) and euthanised (14 and 21 dpi) birds. At necropsy, the severe congestion in lungs and trachea, and pale, swollen kidneys with a visible tubules structure, and/or ureters distended with urates were observed (Figure 2A,B). The intestinal tracts were bloated, filled with yellowish content (Figure 2C). Similar sectional changes were observed in sentinel birds. In older birds (trial 2), at 7 and 14 dpi lung and tracheal congestion, moderate intestinal swelling and kidney oedema were observed. In birds sectioned in 21 dpi only, oedema and renal congestion were observed (Figure 2D).

#### 2.1.3. Histopathological and Immunohistochemistry Findings

**Figure 3:**
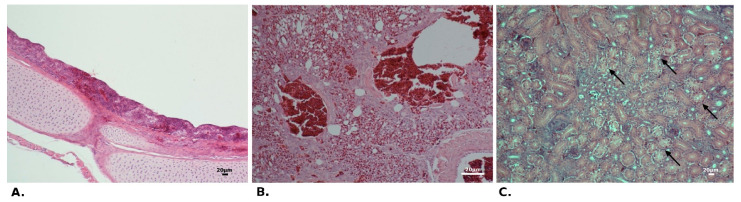
Histopathological changes observed in chickens infected with IBV gammaCoV/Ck/Poland/G052/2016 strain. ((**A**) and (**C**)—trial 1; (**B**)—trial 2) (**A**)—epithelial declination and petechial haemorrhages at the mucosal lining in the trachea of the chicken at 14 dpi; (**B**)—inflammatory cell infiltration and haemorrhage into the parabronchi lumen of the chicken at 7 dpi; and (**C**)—epithelial necrosis in the renal tubules of the chicken at 14 dpi (arrows). The presented scale bar represents 20 µm.

**Figure 4:**
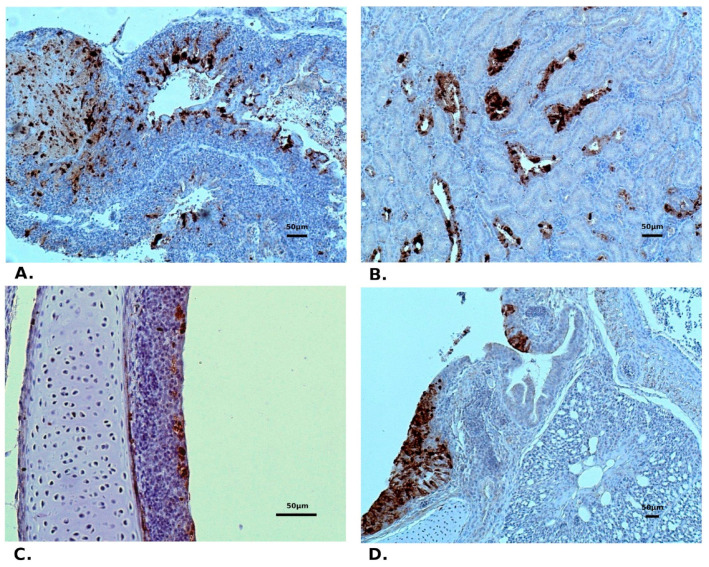
Immunohistochemical (IHC) staining of IBV in tissue samples. Lungs (**A**) and kidney (**B**) of one-day-old chickens at 6 dpi, trachea (**C**) and lungs (**D**) of 3-week-old chickens at 7 dpi. The brown colour indicates antigen immunoreactivity.

In chickens from both trials (one-day-old and three-week-old), severe tracheal lesions were observed in birds between 6 and 14 dpi, including ciliary loss or desquamation of epithelium, mucosal, and submucosal lymphocytic infiltration, as well as mucosal gland atrophy and petechial haemorrhages at the mucosal lining (Figure 3A). At the end of observation (21 dpi) the recovery of the tracheal mucosa was exhibited, although the inflammatory cell infiltration was still observed. Moderate to severe congestion was found in the lungs, with the presence of inflammatory cell infiltration and haemorrhage into the parabronchi lumen during the whole experiment (Figure 3B). Whereas, the microscopic analysis of kidney sections revealed epithelial degeneration or necrosis in the renal tubules with infiltration of lymphoid cells (mainly peritubular) (Figure 3A). No histopathological changes were noted in the duodenum. Microscopic examination of the control birds showed a normal histological structure of the trachea, lungs, duodenum, and kidney, while in two one-day-old birds a slight peritubular lymphocyte infiltration in the kidneys was noted. The presence of IBV antigen in tissues subjected to IHC staining was confirmed mainly in tracheal, lung, and renal samples collected at 6–7 dpi from birds of both age group (Figure 4). No viral antigen or single antigen-positive lymphoid cells were detected in samples taken at 14 and 21 dpi and in the control birds.

#### 2.1.4. Viral Shedding

**Figure 5:**
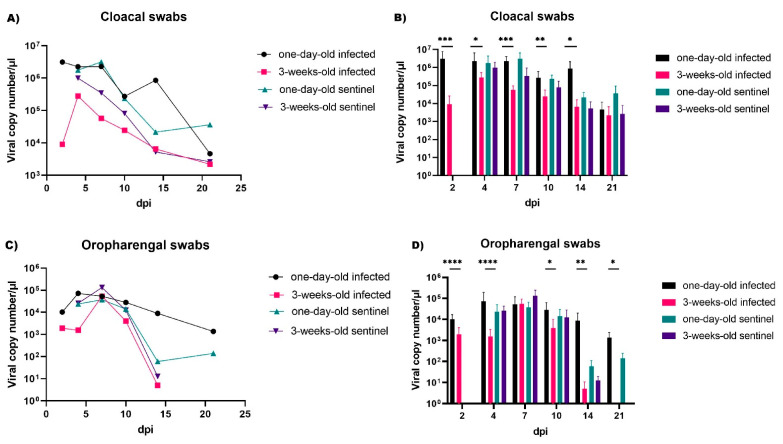
Shedding of IBV gammaCoV/Ck/Poland/G052/2016 strain from the respiratory (**A**,**B**) and digestive tract (**C**,**D**) of infected SPF chickens. The graphs plotted mean copy numbers of viral RNA per µL. Each bar (**B**,**D**) indicates the mean ± SD and statistical significance (* *p* < 0.05, ** *p* < 0.01, *** *p* < 0.001, **** *p* < 0.0001).

The determined viral shedding from the respiratory and digestive tract is shown in Figure 5. Prolonged virus shedding from both tracts was observed in one-day-old chickens in almost all time points, while the viral load in oropharyngeal swabs were lower than those in cloacal swabs. In 3-week-old chickens, the amount of detected virus was lower, especially from the respiratory tract, where the virus was not detected from day 14 onwards. Statistical analysis showed that virus shedding by one-day-old chickens from the digestive tract was significantly higher between two and 14 days post infection (at least tenfold higher at each sampling point) (Figure 5B). Similar results were obtained for oropharyngeal swabs (Figure 5C,D), confirming a higher susceptibility to infection of one-day-old birds. Although on day 7, after infection, the load of virus excreted from respiratory tract in birds of both age groups was at a similar level.

#### 2.1.5. Evaluation of Cross-Protection of Different Vaccination Strategies against IBV gammacov/Ck/Poland/G052/2016 Infection

**Figure 6:**
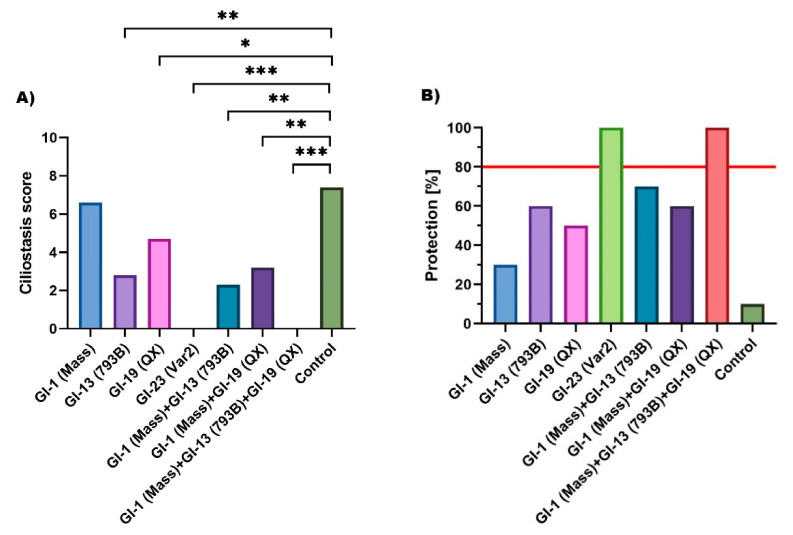
Results of cross-protection of different vaccination programmes for gammaCoV/Ck/Poland/G052/2016 infection. (**A**) Mean ciliostasis score/bird (* *p* < 0.05, ** *p* < 0.01, *** *p* < 0.001). (**B**) Protection (percent of protected birds from total challenged). The red line indicates European Pharmacopeia requirements for cross-protection.

All data regarding experimental groups and study design are presented in Table 1. During 5 days of observation, no clinical symptoms attributed to IBV infection were observed in groups 1–7 and 9, whereas only watery diarrhea was found in birds forming group 8. The detailed results from vaccination-challenge experiments are presented in Table 1 and Figure 6.

The results obtained showed divergent ciliostasis results between birds vaccinated with various type vaccine strains and challenged with IBV Var2 (from 6.6 to 0). Two immunoprophylaxis strategies generated full protection against gammaCoV/Ck/Poland/G052/2016 infection—homologues Var2 vaccine or Mass strain used in one-day-old chickens and boosted by a combination of QX and 793B vaccine. Both programmes resulted in ciliostasis score 0 and 100% protection (Figure 6). In turn, vaccination with Mass strain gives only 30% protection. Any strategy involving additional administration of booster vaccine virus, both QX and 793B strain, resulted in a significant increase in protection to 60 and 70%, respectively. Similar protection was induced by the 793B vaccine administered in the first day of life. However, the QX vaccine given on the first day of life alone induced 50% protection. Not all vaccination regimens meet the European Pharmacopoeia requirements for vaccine efficacy, i.e., at least 80% vaccinated birds should be protected (Figure 6B) [12].

## 3. Discussion

IBV is one of the most important pathogens responsible for economic losses worldwide in the poultry industry. The virus shows variable tissue tropism and virulence properties, and the immunoprophylaxis is the primary measure to control IBV infections [13]. The molecular surveillance of IBV exhibited that GI-23 strains are frequently recognised in recent years in Poland [11]. Field observation showed that the disease outcome was mainly associated with medium respiratory signs and watery diarrhea. In this study, the previously genetically characterised gammaCoV/Ck/Poland/G052/2016 IBV strain was used for in vivo study. First, the virulence properties were assessed using one-day-old and 3-week-old SPF chickens. The results obtained demonstrate that the examined strain is virulent and leads to disease outcome. The clinical signs and histopathological lesions were more severe in younger birds, indicating an age-dependent differences in susceptibility to IBV infection. The virus exhibited mainly respiratory and renal tissue tropism, although the histopathological changes observed in kidneys were more severe (gross renal tubular necrosis). Moreover, in one-day-old birds, infection caused 30% mortality and statistically significantly lower body weight gains (Figure 1). As indicated, virus was shed for an extended period of time via the respiratory and digestive tract. However, the detected viral load was significantly higher from cloacal swabs and in one-day-old chickens throughout the study period. The directly infected birds showed similar viral RNA loads as sentinel birds indicating that the IBV strain is able to spread extensively among chickens. Our findings suggest that the newly emerged GI-23 IBV gammaCoV/Ck/Poland/G052/2016 strain shows the strongest tropism to the renal tract and age-dependent susceptibility of birds to infection. To date, only a few virulence studies have been reported for the IBV GI-23 genotype. Zanaty et al. (2016) reported that infection of one-day-old SPF chickens with the Var2 IBV-Eg/1212B-2012 strain resulted in severe kidney and tracheal lesions and a high mortality rate (50%) [14]. In turn, another IBV GI-23 strain IS/885/00-like, caused tracheal and urinary lesions, and cystic oviduct, but mortality was 2.2% [15]. Both these studies were carried out using one-day-old SFF chickens and broilers and revealed that the virulence properties of GI-23 strains are diverse. Our study reports, for the first time, a comparative study of susceptibility to IBV Var2 infection of different age birds. Both clinical signs and post-mortem lesions were less pronounced in 3-week-old chickens. Similar results were obtained in a study with four nephropathogenic IBV strains, where one-day-old birds were much more susceptible compared to 3-week-old birds [16]. In contrast, in a study by Khanh et al. (2018), infection with the renal QX strain in one- and 30-day-old SPF chickens resulted in a more severe disease course and anatomo-pathological changes in older infected birds (19.5% and 41.3% mortality in younger and older groups, respectively) [17]. However, it should be emphasised that the virulence of IBV strains depends on many factors like virus dose, route of inoculation, age, and breed of the birds or the properties of the strain itself. The observed differences in age susceptibility in relation to our findings may therefore result from the aforementioned factors. The presented results of the in vivo study may be surprising to field veterinarians as they often observe an acute form of disease in 4–6-week-old chickens with high mortality. Although, the clinical course also depends on other variables including environmental and management factors [18] or the presence of concomitant pathogens [19].

Infectious bronchitis virus (IBV) is characterised by high genetic and antigenic variation [3]. This variability is very important, especially with regard to the implementation of an appropriate immunoprophylaxis programme, as vaccination does not always give sufficient cross-protection beyond the virus lineage. The availability of different vaccines on the market gives the possibility to select vaccine strains on the basis of antigenic relatedness with strains circulating in the field. Thus, from a practical point of view, genotyping of IBV field strains is crucial for introduction of an effective immunoprophylaxis. Another aspect of the presented study was the evaluation of protective efficacy of different vaccination strategies against gammaCoV/Ck/Poland/G052/2016 infection. The experimental trial was conducted on commercial broilers and the level of protection was determined using a ciliostasis score according to European Pharmacopeia guidelines [12]. Four vaccines representing Mass (GI-1), 793B (GI-13), QX (GI-19), and Var-2 (GI-23) genotypes were used for immunisation, either alone or in combination (Table 1). The results obtained indicate that the tested vaccine programmes provided different levels of protection (Figure 6B). Furthermore, only two of the seven tested strategies ensured the protection that complies with EP requirements when gammaCoV/Ck/Poland/G052/2016 was challenged. Both, vaccination with Mass after hatching followed by 793B and QX at 14 days of age, and homologous vaccine Var2 resulted with the highest level of cross-protection (100%). Among the tested vaccines alone, next to the homologous vaccine, the best protection was obtained after the administration of the 793B vaccine, which resulted in 60% protection. Vaccination with QX strain conferred 50% protection, while the Mass vaccine only 30%. Understanding the precise mechanism by which IBV antigenicity triggers viral cross-protection still represent key areas of research. In general, a higher sequence homology of the S1 subunit of two strains (e.g., a vaccine and a field strain) should result in a higher cross-protection [3]. However, this is not a general rule, as only a few percent difference in nucleotide similarity of the S1 gene has been shown to cause a significant decrease in cross-protection [20,21]. As previously indicated, the Polish GI-23 strains exhibited high nucleotide homology of the S1 gene with the IS/1494/06 strain used for vaccine production (99.1–99.4%) [8], therefore the obtained high cross-protection with the homologous Var2 vaccine was expected. In turn, the use of three genotype vaccine strains with low nucleotide homology within the HVR3 S1 gene (Mass—74.6%, 793B—75.0%, and QX—73.1%) provided the same protection, confirming the principle formulated by Cook et al. (1999) that the use of vaccines based on different strains extends protection against different genotypes IBV infection [22]. Therefore, before developing a new vaccine, protection against infection with a newly-emerged IBV strain should first be determined using all available vaccines. This has huge implications for laboratory testing, as the diagnostic process is already very complex. It should be added that the recent studies of Jackwood et al. (2020) show that the simultaneous administration of up to four different types of live attenuated vaccines can respond properly without interference and lead adequate protection against infection with each virus type [23]. Taking this into account, it should be assumed that the administration of three tested vaccines would guarantee full protection against Var2 infection earlier in chicken life. To date, several studies have been conducted on cross-protection for Var2 infection [24,25]. Bru et al. (2017) tested three vaccine strains (Mass, D274, and QX) and the best results (70% protection) were obtained with Mass and D274 used in one-day chickens and boosted with QX 14 days later [24]. In turn, Sultan et al. (2019) received 100% protection using Mass in combination with Var2 or Var2 alone [25]. Interestingly, the coupling of Mass strains with 793B resulted in 60% protection, which correspond with the results obtained in our study (70%). Nevertheless, it is difficult to compare the results of these studies as the Var2 strains used for infection are genetically different. Strain used by Sultan et al. Eg/1212B/2012 shows only 82% nucleotide similarity to strain gammaCoV/Ck/Poland/G052/2016 within the HVR3 S1 region (data not published). In turn, the sequence of strain used by Bru et al. (2017) designated as IS/1494/06-like is not available in GenBank database, therefore the nucleotide similarity cannot be assessed.

In conclusion, the current study provides significant insights into the virulence properties and efficacy of different vaccination programmes to infection with newly-emerged GI-23 IBV strains in Poland. Closely phylogenetically-related strains were recently identified in Romania and Germany, indicating the epidemiological threat of this variant in Europe and highlighting the importance of the data obtained. Tropism to the renal and tracheal tissue of the variant strain and age-dependent susceptibility of infected chickens were observed. Moreover, the application of the Mass vaccine in one-day-old chickens boosted by a combination of a QX and 793B vaccine confer 100% protection against European Var2 IBV infection. The results obtained will help in the compilation of an appropriate immunoprophylaxis programme for broiler and layers rearing in the absence of a homologous vaccine approved by the EU authorities on the market.

## 4. Materials and Methods

### 4.1. Ethical Statement

The experimental procedures used in the current study were supervised and approved by the Local Ethical Commission in Lublin (permit no. 32/2016) in agreement with the rules in place in the EU (Directive 2010/63/UE).

### 4.2. Viral Strain

The IBV GI-23 strain gammaCoV/Ck/Poland/G052/2016 previously isolated from a clinical case of IB in 6-week-old broiler chickens and genetically characterised by Lisowska et al. [8] was used to assess the virulence properties and protectotype in animal trials. For the purpose of the present study, the virus was twice propagated in 9-day-old SPF embryonated eggs (Valo BioMedia, Germany) via the chorioallantoic sac route (CAS). The viral titre was determined according to the Reed and Muench method [26] and expressed as an embryo lethal dose (ELD_50_).

### 4.3. Pathogenicity Study in SPF Chickens

The virulence of gammaCoV/Ck/Poland/G052/2016 was assessed using 1-day-old (trial 1) and 3-week-old (trial 2) SPF chickens. In each trial, 20 chickens were used, of which 10 birds were inoculated via the intraocular and intranasal routes with 10^5^ ELD_50_/0.1 mL of virus dose, and 5 chickens served as contact-exposed birds that were inserted the second day after infection. The other 5 chickens were inoculated with saline buffer solution (PBS) and constituted a control group. Birds were separately housed in HEPA-filtered isolators (Montair Andersen B.V., Holland) and daily monitored for 21 days. At 2, 4, 7, 10, 14, and 21 days post-infection (dpi) the oropharyngeal and cloacal swabs were collected. At 7 and 14 dpi two randomly selected birds and at 21 dpi all other birds were euthanised. During necropsy, the pathological lesions were determined and tissue samples (duodenum, trachea, lungs, and kidneys) were collected for histopathological examinations. Birds of all groups were weighed before infection and at the end of the animal trial.

### 4.4. Histopathology and Immunohistochemistry (IHC)

The tracheal, lung, and kidney tissue samples collected during the necropsy were fixed in 10% neutral buffered formalin and processed routinely to paraffin blocks. The sections (4 µm) were stained with haematoxylin and eosin (HE) for histopathological analysis. The same tissue sections were used for immunohistochemistry examinations. Before IHC, staining slides were incubated overnight at 37 °C, then for 30 min at 60 °C. After the deparaffinising procedure, endogenous peroxidase was blocked by placing scraps in 3% solution of hydrogen peroxide (H_2_O_2_) for 10 min, and subsequently heat-induced at 110 °C in citrate buffer (pH 6.0) for 20 min to expose the epitopes. Next, the slides were incubated with TBST buffer (TBS, 0.1% Tween 20) for 5 min. The immunostaining of tissue section was performed using a 1:50 diluted anti-IBV S2 protein monoclonal antibody (Wageningen University, The Netherlands) for 1h at room temperature. After washing with TBST, the sections were treated with anti-mouse secondary antibody, horseradish peroxidase, and DAB chromogen according to producer protocol (Dako Envision+, Dako, UK). Finally, the tissue sections were counterstained with haematoxylin and examined microscopically.

### 4.5. Viral RNA Load by Real-Time RT-qPCR

The oropharyngeal and cloacal swabs were suspended in 1 mL PBS and incubated for 1 h at room temperature and centrifuged at 4 °C, 3500× *g* for 15 min. Total RNA was extracted using the RNeasy Mini Kit (Qiagen, Germany), following the supplier’s instructions. The detection and quantification of the virus load was conducted using the QuantiTect Probe RT-PCR Kit (Qiagen, Germany) as previously described [27].

Viral RNA corresponding to the 5′-UTR fragment of the Var2 IBV G052/2016 strain genome was generated as a viral load standard. Briefly, a 143 bp long amplicon was obtained using the SuperScript III One-Step RT-PCR System with Platinum Taq High Fidelity DNA Polymerase (Invitrogen, Thermo Fischer Scientific, Lithuania) using specific primers [27]. The purified cDNA was cloned into the pJET1.2 vector (Thermo Fischer Scientific, Lithuania) according to the producer’s recommendations and used for the transformation of competent cells of *E. coli*, NZY5α (NZYTech, Portugal). The plasmid DNA was extracted from the bacterial cultures using the NucleoSpin plasmid kit (Macherey-Nagel, Germany) and sequenced to verify its accuracy. The HindIII-digested plasmid was used as a template with the AmpliScribe T7 High Yield Transcription Kit (Lucigen, UK) for in vitro transcription according to the manufacturer’s protocol. The final concentration of RNA transcript was spectrophotometrically determined using NanoPhotometer Pearl (Implen, Germany) and converted to the number of viral copies. A series of tenfold dilutions of plasmid DNA containing 10^6^ copies/µL of viral template was applied in real-time RT-PCR assay to generate standard curves for the determination of the viral load in swab samples. In order to normalise the swab samples, an equal volume of staring material was used for testing. The amounts of IBV RNA were obtained by plotting C_t_ values onto the standard curve.

### 4.6. Evaluation of Cross-Protection of Different Vaccination Strategies against IBV Var2 Infection

Ninety-one-day-old commercial broiler chickens obtained from a commercial producer were equally divided into 9 experimental groups. Groups 1–7 were vaccinated according to the manufacturer’s recommendations and schedule presented in Table 1. Live vaccines representing four different IBV genotypes (Mass, 793B, QX, and Var2) were used for immunoprophylaxis in a study. For the challenge purpose, IBV GI-23 strain gammaCoV/Ck/Poland/G052/2016 in a dose of 10^5^ ELD50/0.1 mL was applied 28 days post the first vaccination. Group 8 served as a challenge control group (not vaccinated, IBV infected), while chickens from group 9 was not vaccinated and not infected and were used as a negative control group. After infection, all groups were monitored daily for 5 days.

### 4.7. Ciliary Activity Inhibition

The level of protection was determined using the ciliostasis test according to the principles described in the European Pharmacopoeia (Monograph 04/2017: 0442, point 2-4-3-1) [12]. Firstly, 10 tracheal rings per chicken at 5 days post challenge was microscopically examined for ciliary activity, which was expressed as 0 when at least 50% of cilia showed vigorous movement, and 1 when the movement was observed in less than 50% of cilia. The maximum possible ciliostasis test result for one bird is 10 points, which means no protection against IBV control infection. Next, the protection level was assessed according to the ciliostasis score (0–10). A chicken was considered as not affected when not fewer than 9 out of 10 rings (ciliostasis score ≤ 1) showed normal ciliary activity. A vaccine complies with requirements of Ph. Eur. for cross-protection if not fewer than 80% of the vaccinated chickens showed normal ciliary activity [12].

### 4.8. Statistical Analysis

Weight gain after IBV Var2 infection was analysed by unpaired *t* test. The data obtained from quantitative analysis of virus load in swab samples and ciliostasis test were analysed by a nonparametric Mann–Whitney test. All analysis were conducted using GraphPad Prism v.9.0.2. The results with *p* < 0.05 were considered as statistically significant.

## Figures and Tables

**Table 1:** Design and results of the cross-protection experimental trial.

Group No.	Vaccination Schedule		Challenge with GI-23 Strainat Day 28	Ciliostasis Score ^1^	% Protection ^2^
	Day 0	Day 14			
1	Nobilis IB Ma5 (GI-1)	none	Yes	6.6	30
2	Cevac IBird (GI-13)	none	Yes	2.8	60
3	Nobilis Primo QX (GI-19)	none	Yes	4.7	50
4	Tabic IB Var206 (GI-23)	none	Yes	0	100
5	Nobilis IB Ma5 (GI-1)	Cevac IBird (GI-13)	Yes	2.3	70
6	Nobilis IB Ma5 (GI-1)	Nobilis Primo QX (GI-19)	Yes	3.2	60
7	Nobilis IB Ma5 (GI-1)	Cevac IBird (GI-13) + Nobilis Primo QX (GI-19)	Yes	0	100
8	none	none	Yes	7.4	10
9	none	none	No	0	-

^1^ Mean ciliostasis score/bird for the 100 tracheal rings examined in group; ^2^ percent of protected birds from total challenged. Bird was considered as protected if not less than 9 out of 10 tracheal rings/bird showed normal ciliary activity.

## Data Availability

The data presented in this study are available on request from the corresponding author.
